# PSG-Audio, a scored polysomnography dataset with simultaneous audio recordings for sleep apnea studies

**DOI:** 10.1038/s41597-021-00977-w

**Published:** 2021-08-03

**Authors:** Georgia Korompili, Anastasia Amfilochiou, Lampros Kokkalas, Stelios A. Mitilineos, Nicolas- Alexander Tatlas, Marios Kouvaras, Emmanouil Kastanakis, Chrysoula Maniou, Stelios M. Potirakis

**Affiliations:** 1 Department of Electrical and Electronic Engineering, University of West Attica, Attica, Greece; 2 Sleep Study Unit, Sismanoglio – Amalia Fleming General Hospital of Athens, Athens, Greece

**Keywords:** Respiratory tract diseases, Biomedical engineering, Electrical and electronic engineering

## Abstract

The sleep apnea syndrome is a chronic condition that affects the quality of life and increases the risk of severe health conditions such as cardiovascular diseases. However, the prevalence of the syndrome in the general population is considered to be heavily underestimated due to the restricted number of people seeking diagnosis, with the leading cause for this being the inconvenience of the current reference standard for apnea diagnosis: Polysomnography. To enhance patients’ awareness of the syndrome, a great endeavour is conducted in the literature. Various home-based apnea detection systems are being developed, profiting from information in a restricted set of polysomnography signals. In particular, breathing sound has been proven highly effective in detecting apneic events during sleep. The development of accurate systems requires multitudinous datasets of audio recordings and polysomnograms. In this work, we provide the first open access dataset, comprising 212 polysomnograms along with synchronized high-quality tracheal and ambient microphone recordings. We envision this dataset to be widely used for the development of home-based apnea detection techniques and frameworks.

## Abstract (summary)

**Table:** 

Measurement(s)	obstructive sleep apnea • tracheal breathing sound • ambient breathing sound
Technology Type(s)	polysomnography • tracheal microphone • ambient microphone
Sample Characteristic - Organism	Homo sapiens

Machine-accessible metadata file describing the reported data: 10.6084/m9.figshare.14611581

## Background & Summary

The sleep apnea syndrome (SAS) is a breathing disorder occurring as a repetitive cessation or severe reduction of breathing, leading to disrupted sleep^1,2^. Although it was noticed even before 1900, under the term “Pickwickian syndrome”, in exceptional cases linked to obesity and hypersomnolence, SAS was recognized as a pathological chronic condition only after 1970^3^. Since then, various studies attempted to determine the SAS relationship with age^4–8^ or gender^4,7,9–12^ and with pathological conditions like obesity^7,13–17^, hypertension^16,18–21^, diabetes mellitus^22–25^, previously reported stroke^26–29^ or cardiovascular diseases^30–34^ and cancer^35^.

The SAS symptoms include daytime fatigue and sleepiness^36,37^, headaches, depression or mood changes^37,38^, disrupted interpersonal relationships^38^, reduced cognitive performance^37,38^ and increased risk of work and vehicle accidents^39–41^. Despite the symptoms’ severity, the awareness of the syndrome remains restricted^42^. The epidemiological studies on apnea prevalence in the general population exhibit increased inconsistency (3–7% for men, 2–5% for women)^43–45^. This is mainly attributed to the medical protocols’ differences^46^. However, the restricted number of people seeking diagnosis also strongly contributes to SAS prevalence underestimation^47^.

The reference standard for diagnosing apnea is Polysomnography (PSG): a study of cardiac, neurological and respiratory signals over a full night sleep in hospital^48^. The PSG inconvenience, along with the requirement for full night hospitalization, discourages patients from seeking diagnosis. Thus, multiple systems for SAS estimation at home have been developed. They provide easier examination and increase patients’ awareness of SAS. The reported studies profit from smartphones’ ubiquity, their connectivity to portable sensors and their computational power, which allows for data processing through built-in applications^49–55^. Breathing sound has been proven highly effective in this endeavour^56,57^. Most studies employ neural networks (NNs)^58–62^, thus, they strongly depend on annotated PSG data and audio recordings via ambient^49^ or contact microphones^59,60^.

The data collection process is crucial for the effectiveness and accuracy of the evolved systems, contributing to both the systems’ development and validation. But data collection is a time-consuming process, requiring a large number of patients to undergo a PSG in hospital. Online available datasets are a convenient alternative to save valuable time and assure feasibility of comparison between different approaches^63–67^. However, to the best of our knowledge, there is currently no available dataset that includes breathing sound recordings simultaneous to PSG. Raw PSG data without contact or ambient microphone recordings are publicly available mainly through the National Sleep Research Resource^65^, referring to sleep studies on the general population^65,68,69^ or on specific subpopulations such as paediatric patients^70,71^, pregnant women^72^ or elderly men^73,74^. While tracheal microphones participate in standard PSG systems, the recordings are usually of poor quality^75^ with low sampling frequency or narrow dynamic range. The lack of standardization protocols with regard to the microphone type, bandwidth and signal compression leads to inconsistent results between the developed methodologies. Even during the implementation of proprietary datasets, sound is rarely recorded simultaneously with PSG, with an exception being the study of Azarbarzin and Moussavi^76^. In audio-oriented studies, sound is usually recorded separately^77^ and reference scoring of apneas relies solely on sound or a restricted number of PSG signals^78^, rendering diagnosis incomparable to PSG findings.

To meet these challenges, we built and provide an open access dataset comprising 212 PSGs with synchronized sound recordings from tracheal and ambient microphones. The data are collected and characterized by the medical team of Sismanoglio – Amalia Fleming General Hospital of Athens, and are open and freely available online {10.11922/sciencedb.00345}^79^. We trust that this dataset will particularly contribute in: (a) comparable studies on the effectiveness of contact and ambient microphones in apnea detection, (b) studies on breathing sound features for specific time detection of apneic episodes and (c) data augmentation of PSG-related studies. The dataset is part of an on-going research study and is expected to be enriched in the following months/years.

Herein, we present the data collection methodology, the emerging ethical issues management, as well as several key dataset statistics since a complete characterization of an open access dataset is highly important in order to support future research findings^80^. A discussion is additionally conducted concerning crucial challenges of this field, particularly the subjectivity of manual respiratory events labelling and the requirements for multitudinous and balanced datasets for developing accurate SAS detection systems.

## Methods

### Ethical issues management

The participation of the patients in the present study is approved by the Local ethics committee of Sismanoglio Hospital and gives rise in several ethical issues that need to be managed following the European Regulation for Personal Data Protection^81^. All patients were asked to give signed consent for participating in the study and agree in audio signal recording during their sleep. They were also asked to agree on the use of all unnamed recorded data for research purposes. The involvement of the health care personnel in the process of the PSG medical examination, providing instruction and help to the patient, requires additional consent for their speech recording at the beginning or during the study.

Data acquired from each patient were stored in the hospital and processed by the health specialists to extract diagnosis. All personal information leading to identification of the participating individuals (names, credentials, contact information, etc.) was removed from the acquired files. An unnamed copy of each PSG study along with the corresponding audio files were stored and further processed to participate in the dataset.

### Signal acquisition and storage

The data were collected from 212 individuals, who visited the Sleep Study Unit of the Sismanoglio – Amalia Fleming General Hospital of Athens for SAS diagnosis. The patients were subjected to a full night sleep study following the standard protocol for split PSG^82^ in which the first part – approximately four (4) hours - is a standard diagnostic PSG while the second part is used for titration to optimal level of pressure, to eliminate apnea events with continuous positive airway pressure (CPAP). The optimum level pressure for all patients following split study protocol is also available in the dataset. If the patient was not suitable for split study, diagnostic PSG was not interrupted. Since the developed dataset is built for studying sleep without CPAP intervention, only the first part of the recorded study was included. A detailed listing of the signals, recorded through the PSG system and the respective sampling frequency for each channel are given in Table 1. PSG channels monitoring and signal acquisition was performed using the Sleepware G3 software.

**Table 1:** Basic properties of the channels included in the EDF files of the dataset.

Channel label	Description	Sampling frequency (Hz)	Physical minimum (a.u.)	Physical maximum (a.u.)	Digital minimum	Digital maximum
‘EEG A1-A2’	Electroencephalogram (reference channel)	200	−313	313	−32768	32767
‘EEG C3-A2’	Electroencephalogram (channel 1)	200	−313	313	−32768	32767
‘EEG C4-A1’	Electroencephalogram (channel 2)	200	−313	313	−32768	32767
‘EOG LOC-A2’	Electroculogram (left eye)	200	−313	313	−32768	32767
‘EOG ROC-A2’	Electroculogram (right eye)	200	−313	313	−32768	32767
‘EMG Chin’	Electromyogram (placed on chin)	200	−78	78	−32768	32767
‘Leg 1’	Leg movement signal (right leg)	200	−78	78	−32768	32767
‘Leg 2’	Leg movement signal (left leg)	200	−78	78	−32768	32767
‘ECG I’	Electrocardiogram	200	−8333	8333	−32768	32767
‘RR’	RR interval in the ECG	10	0	200	0	200
‘Snore’	Contact microphone (placed on trachea)	500	−100	100	−32768	32767
‘Flow Patient’	Thermistor	100	−100	100	−32768	32767
‘Flow Patient’	Pressure cannula	100	−100	100	−32768	32767
‘Effort THO’	Respiratory belt (thoracic volume changes)	100	−100	100	−32768	32767
‘Effort ABD’	Respiratory belt (abdomen volume changes)	100	−100	100	−32768	32767
‘SpO_2_’	Oxymeter	1	0	102.3	0	1023
‘Body’	Position of the body (S = supine, R = right, L = left)	1	0	255	0	255
‘PulseRate’	The pulse rate extracted by the ECG	1	0	255	0	255
‘Tracheal’	High-quality contact microphone (placed on trachea)	48000	−100	100	−32768	32767
‘Microphone’	Ambient microphone (placed ~1 m above patient head)	48000	−100	100	−32768	32767

**Fig. 1:**
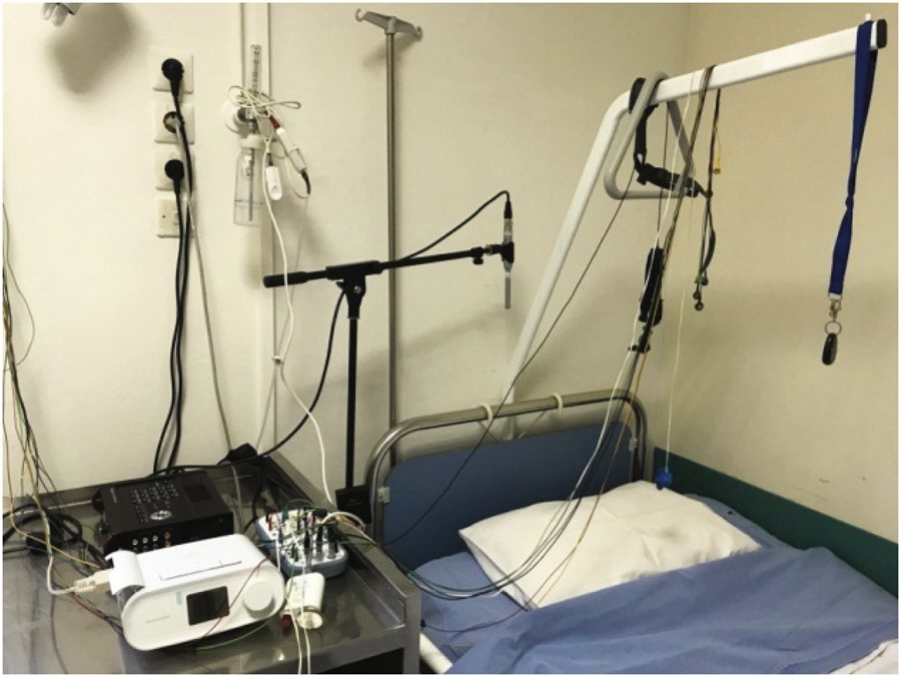
Contact and ambient microphones installed in the sleep study room, along with the multitrack recorder.

Simultaneously with the PSG study a dual channel portable multitrack recorder (Tascam DR-680 MK II) was used in order to acquire and store the audio signals from two high-quality microphones: (a) a contact microphone (Clockaudio CTH100) placed on the trachea of the patient and (b) an ultra-linear measurement condenser microphone (Behringer ECM8000) placed approximately 1 m above the patient’s bed, over the head position (Fig. 1). Both sound signals are sampled at 48 kHz and stored in an SD card as 24-bit uncompressed Waveform Audio Format (.wav) files. The contact electret microphone (input impedance: 900 Ω, passband: 350 Hz – 8 kHz) acquires only the neck vibrations while it is completely insensitive to environmental noise. The ambient condenser microphone is of electret type, omnidirectional, with an input impedance of 200 Ω and a flat frequency response in the range of 15 Hz–20 kHz.

### Medical data annotation process and diagnosis extraction

Medical characterization of each particular PSG study is performed by the health specialists of the Sleep Study Unit of the Sismanoglio – Amalia Fleming General Hospital of Athens. For each patient, sleep stages and apnea events are scored by two specialists: a certified technician performs first level scoring and a 30-year-experienced and certified doctor performs final scoring, with verification of the true positive annotated events and addition of missed events. The process of scoring between the specialists is not blind, however, the followed protocol assures increased accuracy. Inter-observer agreement was evaluated in the past, maximizing the provided accuracy through the followed process, while further examination of the inter- and intra- observer error is beyond the goals of the current project. Additionally, we developed an algorithm that quantifies the decrease in the flow rate amplitude within each scored event to validate data and assure high accuracy in the true positive events annotation. The algorithm details and the results are presented in Section 3.5.

The scoring of sleep stages during the total sleep time (TST) relies on the general instructions for sleep stage labelling^1,83^. The detection of apnea/hypopnea events, during the recorded sleeping hours, was performed manually by simultaneous observation of all channels of the PSG system, according to the general criteria for apnea episode scoring. Audio recordings were not included in the diagnostic process. The final diagnosis concerning the categorization of the patient in one of the reported apnea severity cases: “Severe”, “Moderate”, “Mild Apnea” and “Normal” was extracted through the Apnea/Hypopnea Index (AHI). The AHI is defined as the ratio of the total count of apneic episodes in the entire sleep study over the TST in hours [1], which results in the mean count of apneic events per sleeping hour. Up to 5 apnea/hypopnea episodes per hour classify the subject in the case of “Normal breathing” during sleep while higher values indicate a gradually increasing severity of SAS (5 episodes/h ≤ AHI < 15 episodes/h: “Mild Apnea”, 15 episodes/h ≤ AHI < 30 episodes/h: “Moderate Apnea”, 30 episodes/h ≤ AHI: “Severe Apnea”)^1,2^.

### Signals synchronization

A critical step is the synchronization of the acquired audio signals with the PSG signals derived from separately activated systems. The synchronization algorithm employs the tracheal sound recorded from a separate, low quality, contact microphone of the PSG system (channel label “Snore”) – sampling frequency: 500 Hz and bit depth: 16-bits. The two acquired tracheal sound signals were synchronized by extracting the signal envelope with Hilbert transform. Prior to this step, it was necessary to reduce the sampling rate of the high-quality tracheal signal so that the two signals have a common sampling frequency of 500 Hz. The cross correlation of the two signal envelopes is extracted and the delay in the activation of the two systems is estimated by the time difference value that maximizes cross correlation. To accurately determine this delay, we examine at least 10 min of the signals, while the supervisors of the sleep study are given the instruction to activate the two systems with the minimum possible delay, most frequently below 30 s. Manual observation of the signals results in the estimation of the error in synchronization and the rejection of those PSG studies that exhibit an error higher than 2 s in this step. Thus, among a total number of 240 patients that underwent PSG study between April 2019 and July 2020, 28 patients were rejected from the herein presented dataset as they exhibit low quality audio or PSG recordings – mainly due to the dislocation of one or more sensors – and the corresponding signals could not be accurately synchronized.

## Data Records

The storage of polysomnographic and breathing sound data was performed by employing the European Data Format (EDF) common in medical data storage and transfer^84^. The EDF files contain the channels listed in Table 1, with the corresponding sampling frequency and digital and physical maximum and minimum values. While the PSG data were retrieved from equivalent EDF files and remain unchanged, the additional audio data were retrieved from uncompressed WAV files and stored in the EDF files under the channel names “Tracheal” and “Microphone”, corresponding to the tracheal sound signal and the ambient microphone signal respectively. The import of the separately recorded high-quality audio data (“Tracheal” and “Microphone”) was performed by using zero padding policy for the missing parts of the signals, as a result of the synchronization process. EDF is selected due to its popularity in medical data storage and transform, despite the fact that, for the audio signal, this format results in a reduction of the bit depth. Indeed, the most common version of EDF files requires storage in 16-bit while the original WAV files were stored in 24-bit depth. Alternatively, BioSemi Data Format (BDF) files could be used, which is a 24-bit version of EDF files; however, this option was rejected due to its still restricted popularity and the existence of fewer software platforms that support it. The conversion of the initially acquired high-quality audio recordings from 24 to 16-bit depth is performed by neglecting the least significant bits of each sample value. The statistical absolute error due to this conversion presents an average value of 7.6291 · 10^−6^ while the maximum value does not exceed 1.5259 · 10^−5^. These values impose minor quality reduction in the audio recordings that is not expected to affect the studies related to breathing or snoring sound properties. The EDF files corresponding to each patient study are cropped in parts of 1 h duration each, to facilitate handling. Each patient was labelled with a unique representative patient number (range 993–1496).

In the dataset, additional .rml files are responsible for all annotations corresponding to each patient. These files include the sleeping stages and the labelled events. The annotated events are characterized by the family in which they belong (“respiratory”, “neurological”, “limb activity related”, “nasal” and “cardiac”) and the type, which is related to each event family according to Table 2. In particular, the “respiratory” episodes, which are the main concern of this report, include among others all apnea related episodes of specific type: “Obstructive Apnea”, “Central Apnea”, “Mixed Apnea” and “Hypopnea”. Additionally, the.rml files contain all annotated episodes of relative oxygen desaturation and arousal events. Concerning the patients’ data, the information on the age and gender of each participating individual were kept in the corresponding.rml files.

**Table 2:** Annotated events family and corresponding types.

Event Family	Specific types
Respiratory	‘Obstructive Apnea’/‘Central Apnea’/‘Mixed Apnea’/‘Hypopnea’/‘Cheyne Stokes Respiration’/‘Periodic Respiration’/‘Respiratory effort- related arousal (RERA)’
Neurological	‘Arousal’
Limb	‘Leg Movement’/‘Alternating Leg Muscle Activation’/‘Hypnagogic Foot Tremor’/‘Excessive Freagmentary Myoclonus’/‘Rythmic Movement Disorder’
Nasal	‘Snore’
Cardiac	‘Bradycardia’/‘Tachycardia’/‘Long RR’/‘Ptt Drop’/‘Heart rate Drop’/‘Heart Rate Rise’/‘Asystole’/‘Sinus Tachycardia’/‘Narrow Complex Tachycardia’/‘Wide Complex Tachycardia’/‘Atrial Fibrilation’
SpO_2_	‘Relative Desaturation’/‘Absolute Desaturation’

All data are stored in an open access dataset, available for free download here: 10.11922/sciencedb.00345^79^.

## Technical Validation

### Basic statistical features on the divergence of patients participating in the dataset

**Fig. 2:**
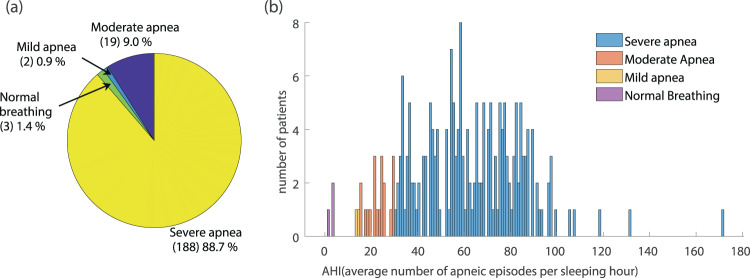
Dataset statistics on patients’ apnea syndrome severity. (**a**) Patients’ distribution among the classes of different apnea severity. (**b**) The distribution of AHI value in all subjects participating in the dataset with labeling of the corresponding apnea severity class.

**Fig. 3:**
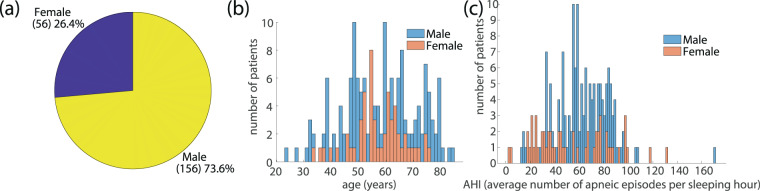
Dataset variability with regards to the patients’ age and gender. (**a**) Gender distribution and (**b**) age distribution examined separately for men and women for all subjects participating in the dataset. (**c**) AHI distribution separately for the male and female participants.

A major issue in the use of polysomnographic data in related studies and systems development is the balancing between different categories of patients that are included in the used dataset. The divergence of the participating subjects with regards to factors such as the gender, the age and the final diagnosis of SAS severity may noticeably alter the features of recorded breathing and snoring sound and the episodes properties such as the duration of each episode. The PSG is usually prescribed to patients who complain about excessive sleepiness during daytime or loud snoring during sleep, symptoms that are strongly related to the presence of SAS. As expected, the majority of the participants (88.7%) belong to the group of “Severe Apnea” while the percentage of “normal” cases is restricted, not exceeding 1.4% of all diagnosed individuals (Fig. 2a). Taking into account the increased risk of SAS in male population, the gender classification of the participating subjects is strongly imbalanced, with male population representing 76% of the entire dataset (Fig. 3a). The age distribution of patients ranges from 34 to 76 years for women and 23 to 85 years for men. The mean values are 57.2 and 57.9 years for women and men respectively, with different ages equally distributed in men and women (Fig. 3b).

The age distribution of the total population participating in the dataset exhibits significant similarities between the different groups of apnea severity classes (see Supplementary Fig. 1) despite the existence of imbalanced data among them. It is also interesting that the distribution of AHI extends over approximately the same range for both male and female individuals (Fig. 3c). These statistics assure that although the dataset is subjected to severe unbalancing between the different groups, the dataset information concerning apnea/hyponea episodes covers a wide range of AHI – consequently all SAS severity classes – and a wide range of ages. It is reminded that all statistical measures presented here should not be considered as epidemiological data but only as features indicative of the dataset’s balancing.

### The annotated apnea related episodes

**Fig. 4:**
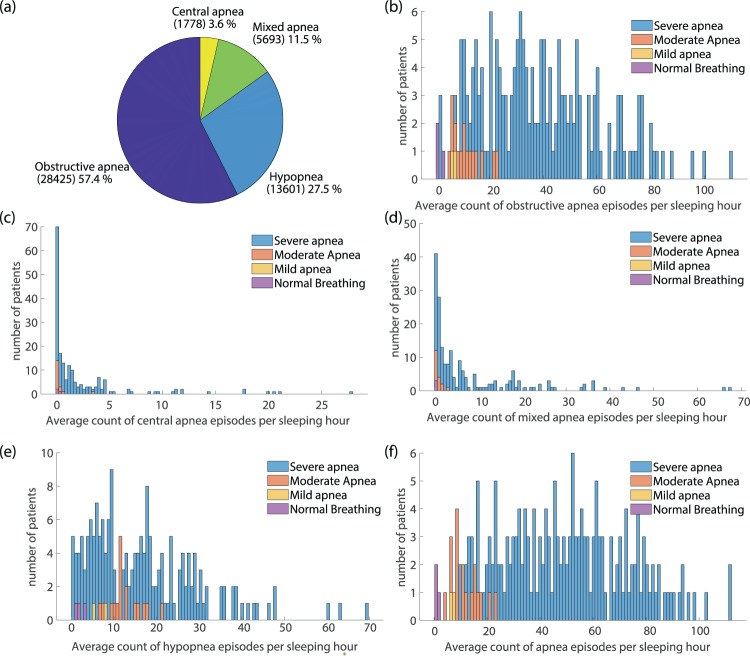
Dataset variability with regards to the scored apnea/hypopnea episodes. (**a**) Frequency of appearance of different types of apnea/hypopnea episodes in the provided data. The distribution of (**b**) obstructive apnea, (**c**) central apnea (**d**) mixed apnea and (**e**) hypopnea events per sleeping hour for patients belonging in different SAS severity class. (**e**) and (**f**): comparison of Apnea Index (AI) and Hypopnea Index (HI) as separate indicators contributing to the final overall characterization of the patient with regards to SAS severity.

The labelled apnea/hypopnea events belong to the “Respiratory” family and were further subcategorized in the corresponding types of “Obstructive Apnea”, “Central Apnea”, “Mixed Apnea” and “Hypopnea”. Although the software in use for manual observation and annotation of data (Sleepware G3) allows for labelling of subtypes of hypopnea, the latest protocol for apnea scoring^1^, followed in this work, suggests the subdivision of hypopnea events into the cause-related types (Obstructive and Central) to be avoided^85,86^. The majority (99.94%) of all annotated respiratory events (total sum 49525 episodes) were specific types of Apnea (35896 events) or Hypopnea (13601 episodes). As expected, the obstructive sleep apnea episodes dominate in frequency of appearance among all annotated apneic episodes (57.4%), while central apnea events represent only 3.6% of the total count of labelled episodes (Fig. 4a).

The criteria for labelling all types of different apnea episodes are clearly defined in the protocol for sleep apnea scoring^1^. These criteria refer to air flow signals – measured through pressure drop and air thermal changes close to patient’s nose – or to the thoracoabdominal movements representing the breathing effort. The oxygen relative desaturation and possible arousal – indicated by the corresponding neurological signals – are employed as additional factors contributing to the safe identification of apneic episodes. The employed criteria are equal for all patients. The sound signal does not participate in the diagnosis process. However, the research approaches relying on snoring and breathing sound recordings to perform apnea detection, frequently discuss the variation of sound features between patients with different SAS severity. An apneic episode may significantly differ in terms of sound characteristics for a mild snorer compared to a heavy snorer, while snoring characteristics are strongly related to the presence of SAS. Therefore, the distribution of annotated episodes in patients belonging to a different SAS severity class is considered an important factor, indicative of the expected variability of sound features. Figure 4(b–e) summarizes the distribution of the main four categories of apneic events (“Obstructive”, “Central”, “Mixed” and “Hypopnea”) per sleeping hour in relation to the patients’ overall diagnosis (SAS severity class: “Severe”, “Moderate”, “Mild” and “Normal”). As expected by the prevalence of central apnea syndrome, the frequency of central and mixed apnea episodes is concentrated in the range between 0–5 and 0–20 apneas per sleeping hour, respectively, independently of the severity group in which the patient is classified. The obstructive apnea events represent the major contributor to the final AHI and consequently to the overall diagnosis for the patient^87,88^, with the variation in the frequency of appearance resembling the AHI distribution. The hypopnea events of this dataset are particularly frequent in the case of moderately apneic individuals, though such a deduction should also take into account the restricted number of moderate apneic subjects participating in the dataset and it requires further investigation. In Fig. 4e,f a comparison of the Apnea Index (AI) and Hypopnea Index (HI) is attempted. The AI is extracted as the sum of all types of apnea events per sleeping hour, while HI includes only the hypopnea episodes present per hour of sleep. Except for the recent tendency towards separate interpretation of these indices for more accurate diagnosis of SAS severity, the aforementioned comparison clearly indicates that apnea events contribute mostly to the overall SAS severity estimation for this dataset. This might be a useful aspect to consider in the development of sound-based systems for SAS detection, taking into account that the air flowing, through the completely or partially collapsed upper airway system of the patient respectively, must result in significantly different sound characteristics.

**Fig. 5:**
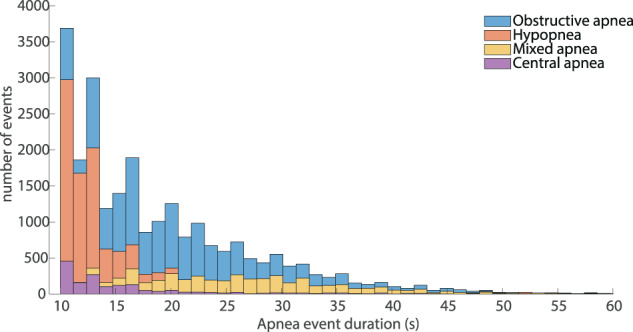
Distribution of the duration of events labeled in the dataset. The distribution is given separately for the main four categories of apnea/hypopnea events (“Obstructive”, “Central”, “Mixed” and “Hypopnea”).

Additionally, Fig. 5 illustrates the distribution of episodes’ duration with regard to the types of apnea/hypopnea. The duration of apnea/hypopnea episodes is believed to represent a particularly interesting factor in the process of sleep study interpretation, suggested by multiple researchers as a separate index to be measured. It is believed that the duration of an apneic episode highly correlates with the effects of relative oxygen desaturation and probably with significant hypoxemia^89^. In the provided dataset, the duration of labelled episodes ranges from 10 s (minimum duration of an acceptable apneic episode) to 128.5 s corresponding to a specific “mixed” apnea event. The distribution of apneic events duration, studied separately for the four main types of apnea, indicates that “mixed” apneas exhibit a significantly higher mean duration (24.6457 s) compared to all other types − 19.0656 s, 16.1016 s and 16.162 s mean duration for “obstructive”, “central” and “hypopnea” events, respectively. The distributions of the mean and maximum event duration per patient do not exhibit significant differences that could classify patients into the four SAS severity categories (see Supplementary Fig. 2) with the longer apneas being present in “Severe” apnea patients and the shorter in “Normal breathing” subjects.

### Sleeping vs. recording time and intra-night AHI variability in the collected PSGs

**Fig. 6:**
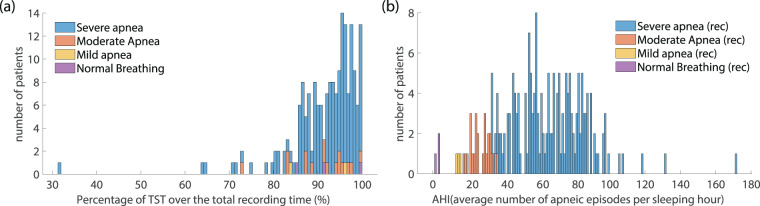
Statistics of the dataset with respect to the scored TST and the total recording time for each patient. (**a**) Difference between the TST and the total recording time, expressed as a percentage over the total recording hours. (**b**) The estimated AHI when the total recording time is taken into account instead of the TST, for each patient. The provided AHI should be used as reference value for the development of home-based systems that do not perform detection of the sleep stages.

The development of home-based systems aims at patients’ convenience; therefore, a restricted number of sensors are employed. A major argument questioning the provided accuracy by home-based systems in comparison to the gold standard of PSG study is the inability of these systems to count the TST of the patient. Assuming that the total recording time can replace the TST, home-based apnea detectors may result in a severe underestimation of the AHI. In the provided dataset, the TST of the patients – identified through the PSG neurological channels – is compared to the total recording time. For the majority of the participating individuals (95.05%) the TST was more than 80% of the recording time, while for approximately 70% of them the difference between the two values did not exceed 10% (Fig. 6a). These results are practically independent from the severity class.

The impact on the AHI and SAS severity estimation can be calculated through the comparison of the AHI – derived by the TST of each patient – and the hypothetical SAS severity estimation based on the ratio of the number of apneas/hypopneas over the total recording time of each individual study. The results, illustrated in Fig. 6b,indicate that only 8 out of 212 patients would have been misdiagnosed. In particular, the change in classification appears mainly as a demotion from “Severe” to “Moderate” apnea classes.

**Fig. 7:**
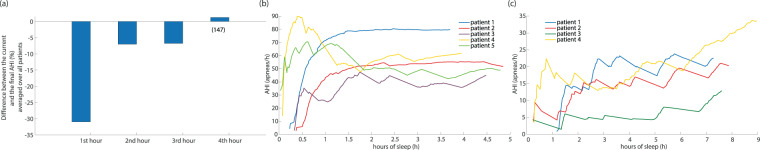
Dataset description with respect to the AHI intra-night variability. (**a**) The mean (averaged over all patients) difference between the current and final AHI for the first four hours of sleep. Only the patients with at least 3 complete hours of sleep (206 out of 212) participate in the extracted statistical values, while the average difference between the 4th hour AHI and the final AHI is extracted by 147 patients that have completed the 4th hour of sleep. (**b**) The intra-night variation of AHI for 5 patients following a split PSG study protocol. (**c**) The intra-night variation of AHI for 4 patients that have completed at least 7 hours of recordings without CPAP.

In the same context, intra-night variability of the AHI can noticeably alter the final diagnosis in case only a few hours of sleep are taken into account. Recent publications, in the field of AHI interpretation, prove the significant variability of the index through the night and particularly the increase of obstructive apnea events frequency towards morning^90^. More precisely, the researchers suggest that the use of the highest AHI met through a time frame of 2 hours could be beneficial in reaching high level of accuracy in the SAS severity diagnosis. In the dataset provided herein, the intra-night variations of AHI are presented though (a) the study of the first 3 hours of sleep and (b) the difference between the current and the final AHI – derived as the averaged frequency of apneic events over the TST. In this particular statistical value, representing the dataset, we excluded the patients that did not complete a minimum duration of 3 h of recordings in the split-study protocol. Thus, only 177 patients appear in the plot of Fig. 7a. It is indicated that the mean difference between the current and total AHI (averaged for all subjects) gradually decreases reaching ~7% after the second hour of sleep (Fig. 7a). However, this difference, studied particularly for each patient, proves the existence of cases where the AHI of the first 3 hours of sleep is significantly lower than the final AHI (see Supplementary Fig. 3). By studying some specific cases of patients, we noticed the presence of a large number among them, in which the AHI remains stable after the 2nd sleeping hour, with the variations in these first 2 hours of sleep either overestimating or underestimating SAS severity (Fig. 7b). By studying the cases of patients subjected to a full night sleep protocol (total recording time at least equal to 7 h) we noticed that the variation of AHI may be important, leading to a final diagnosis change even within the last hour of sleep (Fig. 7c). It is therefore concluded that longer recordings can cover all different cases and lead to more accurate diagnosis, while it might be beneficial for home-based systems to rely on the recordings after the first two hours of sleep where the variations of AHI seem to reduce with respect to the final index.

### Additional PSG channels: the SpO_2_ and arousal events used in apneic/hypopneic events’ detection

**Fig. 8:**
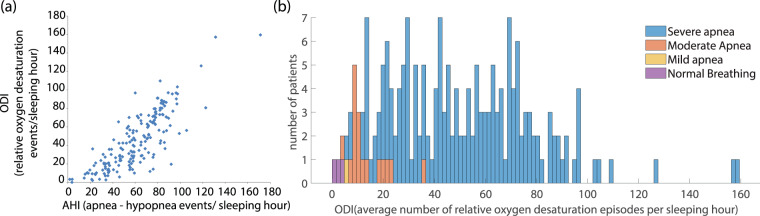
Dataset statistics with respect to the scored ODI per patient. (**a**) Relationship between the ODI and the AHI for all patients in the dataset. While the ascending behavior of the ODI with the increase of the AHI is obvious, the goodness of fit with a linear model expressed by 4 factors is SSE: 5.881e + 04, R-square: 0.6801, Adjusted R-square: 0.6786, RMSE: 16.73. (**b**) The distribution of ODI for patients belonging to different SAS severity class.

Oxygen desaturation during sleep is of crucial importance since it indicates the degree of hypoxemia^91,92^. The Oxygen Desaturation Index (ODI) is defined as the average number of oxygen desaturation episodes per sleeping hour and it strongly correlates with the AHI of a patient^93,94^. In the dataset reported herein, the ODI exhibits an ascending behaviour when AHI increases (Fig. 8a). However, the indicated factors quantifying the goodness of fit with a linear relationship between the two indices (sum of squares due to error - SSE: 5.881e + 04, R-square: 0.6801, Adjusted R-square: 0.6786, root mean squared error - RMSE: 16.73) prove that there are cases of patients exhibiting low ODI while their AHI classifies them in the upper groups of SAS severity (“severe” or “moderate”). Based on the literature, the ODI is often studied, either along with AHI or separately, for the diagnosis of SAS severity^95^. In Fig. 8b,we illustrate the ODI distribution for each class of SAS severity for all patients of this dataset. Ιt is clearly depicted that the vast majority of patients characterized by severe apnea present an increased ODI while those with moderate or mild apnea exhibit a much lower ODI.

The association of ODI with AHI is partially explained by the fact that an apneic episode is frequently (but not always) followed by an episode of relative decrease of oxygen saturation; an oxygen desaturation event is defined as an episode of at least 3% reduction in the oxygen saturation level. In many available systems, these events have been employed as indicative of the preceding apneic event^96^. Herein, we report basic statistical values of the oxygen desaturation events that are linked to a specific event of apnea/hypopnea (of any type: “Obstructive”, “Central”, “Mixed apnea” or “Hypopnea”). The criterion for the association of the two events is not clearly determined in the literature. Some studies have used the criterion that the oxygen desaturation occurs within a frame of 60 s with reference to the onset of the associated apnea/hypopnea episode^97^. We follow the same criterion resulting in 31178 apnea/hypopnea events – among the sum of 49497 events annotated in this dataset – followed by a relative oxygen desaturation episode, thus a percentage of 62.99% of all labelled events. Particularly, 65.63% of the “obstructive” apneas, 60.24% of “central” apneas and 70.88% of “mixed” apneas were followed by a desaturation event. A lower percentage of “hypopneas” is accompanied by an episode of oxygen saturation drop (only 54.53%).

**Fig. 9:**
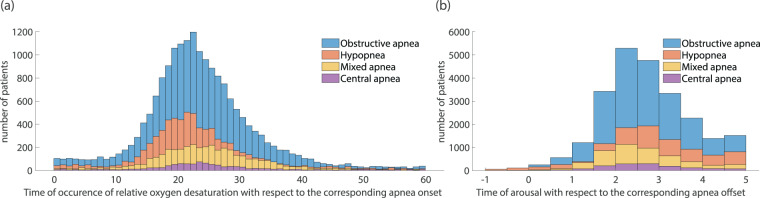
Dataset statistics with respect to the annotated relative oxygen desaturation events and arousals. (**a**) Distribution of time of occurrence of oxygen desaturation events with respect to the associated apnea related episode onset for the main types of apnea/hypopnea (Obstructive, Central, Mixed apnea and Hypopnea). (**b**) Distribution of the time of arousal occurrence with respect to the end time of the associated apnea, illustrated separately for each type of apnea.

Among the SpO_2_ episodes that are associated with a specific apnea/hypopnea event, 94.5% appear with a delay of at least 10 s with reference to the onset of the event. From the distribution of the time delay in the occurrence of oxygen desaturation in the different types of apneic events it is clear that negligible differences appear between the different types of apnea/hypopnea (Fig. 9a). Moreover, for this dataset the mean delay in an SpO_2_ event occurrence does not seem to correlate with the SAS severity class (see Supplementary Fig. 4), despite what is recently discussed in literature^97^. The statistical features presented in this section should be seen through the prism of the imbalanced number of subjects participating in each SAS severity group but also of the fact that the manual process for apneic events labelling was performed independently from the oxygen desaturation events’ scoring.

In the same context, the arousals associated with the presence of apnea/hypopnea events have been studied. Regarded as an immediate consequence of an apnea, an arousal accompanies an apneic event and results in cessation of sleep. In this dataset, a percentage of 80.9% (40042 events) of all annotated apneas – of any type – are followed by an immediate arousal event, occurring within a time frame of 5 s after the end of the apnea/hypopnea episode. When studying separately each type of apnea: “obstructive”, “mixed” and “central” apneas present a percentage of 84.83%, 86.53% and 76.55% respectively, while “hypopneas” exhibit a lower percentage of 70.89%. Based on the appropriate statistical values, we can conclude that for the majority (159 out of 212) of the patients – regardless the SAS severity of their final diagnosis – more than 80% of apneic episodes are accompanied by an immediate arousal (see Supplementary Fig. 5), while the time of occurrence of the arousal is by average located 2.5 s after the end of the apneic episode. The arousal onset time does not seem to depend on the type of preceding apnea, since the distribution of the time of occurrence for each type separately indicates similar mean values for all types (Fig. 9b).

### Subjectivity of manual labelling of apnea/hypopnea events: How significant is the human induced error in SAS severity estimation

With the scoring of the apneic events being performed manually by the simultaneous observation of multiple channel, we do expect that a human error is induced in the dataset scoring^98^. This error should be mainly attributed to the inability of the doctor to precisely determine the degree of air flow reduction by visual observation of the flow rate signals (signal label: ‘Flow Patient’ corresponding to a thermistor and a pressure cannula sensor). For the case of a candidate apneic/hypopneic episode presenting a moderate reduction (close to 30%) in the flow rate, the doctors are forced to label the event as positive apneic to eliminate the risk of false negative diagnosis of the SAS severity of the patient. Consequently, an increased number of false positive annotated apneas are anticipated in the dataset.

In order to examine the degree of human induced error, we developed a simple algorithm that automatically checks the compliance of the annotated events’ characteristics with the recommended criteria for apnea scoring^1^. The rule being followed requires, for any respiratory event, a reduction in both flow rate sensors at least equal to 30% of the amplitude in the previous state of normal breathing. It is also mandatory for an annotated apnea event to exhibit a dominant frequency within the range of normal breathing rate in order to be excluded from the positive apneas list. A normal breath lasts for approximately 3–5 s. Thus a range of 0.16–0.4 Hz was selected for the breathing rate rule. The algorithmic process, for flow rate amplitude reduction, extracts the signal envelop through Hilbert transformation, in a way that restricted in time changes – less than 3 s in duration – are ignored. By examining a 5 s time frame prior and after the annotated event, we extracted the maximum flow rate amplitude corresponding to normal breathing. The comparison of this normal breathing amplitude with the minimum amplitude detected within the annotated event determines whether the candidate episode should be accepted as positively annotated or should be rejected as apnea-negative.

**Fig. 10:**
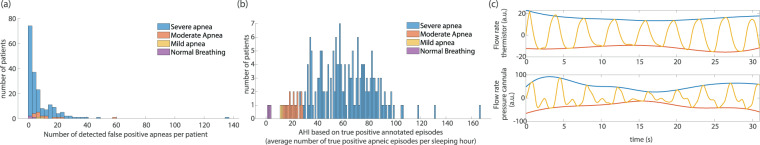
False positive detections due to manual apnea/hypopnea events scoring. (**a**) Distribution of the number of detected false positive annotated apneas per patient along with the characterization of SAS diagnosis. (**b**) Distribution of AHI extracted by the true positive apneas per sleeping hour for all patients along with the originally derived diagnosis for SAS severity. (**c**) Flow rate for an example of a rejected annotated apnea. Reduction in the flow rate within the annotated episode is insufficient to categorize this episode in one of the types of apnea.

The aforementioned process resulted in the rejection of 1711 false positive annotated apneas among the total sum of 49497 events (3.46%). It is important to note that the number of falsely positive annotated apneas does not seem to correlate with the overall diagnosis of the patient (Fig. 10a). The impact of false positive apneas on the SAS severity estimation is minor, with only 4.24% (9 out of the total sum 212) of the patients receiving an overestimated classification of their state of apnea severity. The distribution of AHI extracted by the true positive annotated apnea/hypopnea events along with the delivered to patient SAS severity diagnosis is illustrated in Fig. 10b.

Among the identified false positive apneas the majority belongs to the type of “hypopnea” (1448 events), 257 were classified to “obstructive” apnea, only 5 events were of type “mixed” apnea and 1 event of type “central”. This particularly illustrates the shortcoming in the quantification of the reduction in the airflow by simple observation of the signals by the doctor.

Two major issues concerning interpretation of flow rate signals have been extensively discussed in the literature. In particular, it is reported that the use of only the nasal pressure sensor could lead to an overestimation of apnea events since the air flow through mouth is entirely ignored^99^. It is also reported that pressure sensors are non-linear therefore their use frequently leads to an overestimation of the flow signal amplitude and an erroneously overdetection of hypopneic events^99,100^. The second important aspect affecting interpretation of airflow-related PSG channels is the accuracy limitations of thermistors. They are reported to underestimate flow reduction, through specific flow pattern studies simulating breathing process^101^. This non-linearity of thermal sensors may lead to a severe under-detection of apneic or hypopneic events^90,92^. Though the aforementioned issues still remain under discussion in the literature, the criteria set in this work, for false positive detection counting, concerned both channels with the requirement to exhibit simultaneously a noticeable reduction of amplitude. In the case where only one of the employed sensors monitors a flow reduction, the event was accounted for false positive detection. An exception in this process appears when the signal exhibits very low amplitude, considered as noise. In that case, the corresponding channel was entirely excluded from the investigation, since the sensor could be temporarily dislocated and the corresponding results could be misleading.

While the rejection of the detected false positive annotated apneas slightly alter the final diagnosis for each patient, the extraction of a list of true positive apneas may be valuable for the development of highly accurate automatic systems for SAS estimation. Thus, we opted for the inclusion of additional events annotation (.rml) files in the dataset named with the prefix “clean” for use in comparative studies and particularly in studies aiming at the development of automatic SAS estimation systems based on breathing sound.

## Supplementary information

Supplementary Information

## Data Availability

In this study we used Sleepware G3 software for all PSG data acquisition and events annotation. The software is provided by Philips Inc. The custom code used in this work refers to: (a) the algorithm for the synchronization of audio recordings with the PSG signals, so that the information of all annotated episodes and sleep stages can be accurately transferred to the audio signals as well and (b) the detection of false positive apnea/hypopnea events due to the inability of the doctors to precisely quantify the reduction in the airflow amplitude measured through the flow rate channels of the PSG (thermistor and pressure cannula sensors). All custom code developed for this study is available online (“code.rar” file) along with the files of the dataset^79^.
