# Supplementary material for: PSG-Audio, a scored polysomnography dataset with simultaneous audio recordings for sleep apnea studies
Source: Sci Data. 2021 Aug 3;8:197. doi: 10.1038/s41597-021-00977-w (PMC8333307; doi:10.1038/s41597-021-00977-w)
Supplement: Supplementary file 1 — Supplementary Information [file 41597_2021_977_MOESM1_ESM.docx]

SUPPLEMENTARY INFORMATION

Table of Contents

[1. Age distribution vs SAS severity 1](#_Toc72346340)

[2. Maximum and average apneic event duration per patient vs. SAS severity 1](#_Toc72346341)

[3. Intra-night variation of AHI for the 1^st^, 2^nd^ and 3^rd^ hour of sleep 2](#_Toc72346342)

[4. Apneas/hypopneas followed by oxygen desaturation event 2](#_Toc72346343)

[5. Apneas/hypopneas followed by an arousal 3](#_Toc72346344)

# Age distribution vs SAS severity

The distribution of the age of patients along with the SAS severity diagnosis they received at the end of the PSG study has been also studied as a factor determining the balancing of the provided data. The information provided by the following histogram should be studied through the prism of higher percentage of patients diagnosed as severely suffering from SAS.


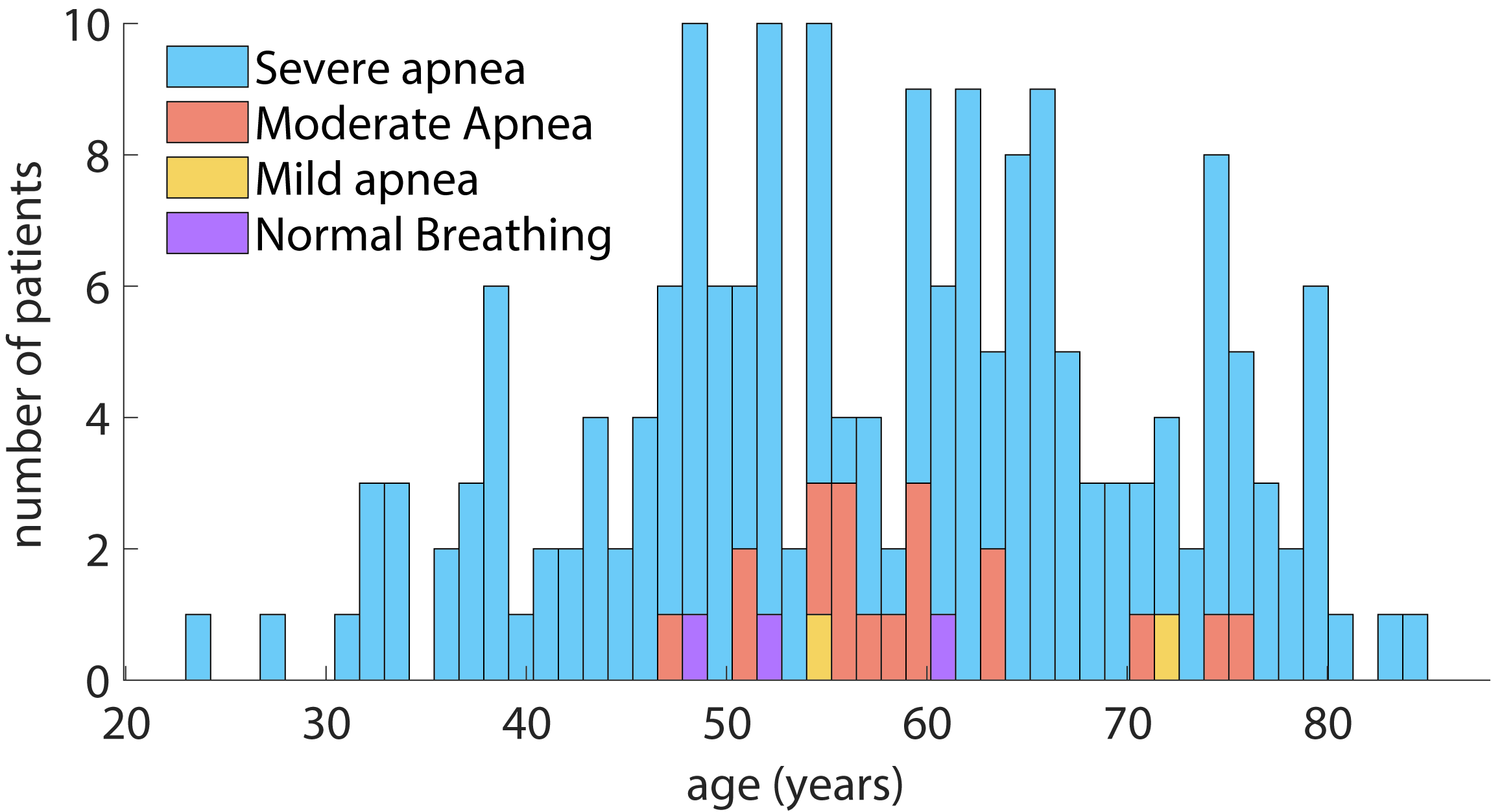


Fig. 1_supp The distribution of patients’ age with respect to their finally diagnosis for SAS severity.

# Maximum and average apneic event duration per patient vs. SAS severity

The duration of all apnea/hypopnea events scored in the dataset have been studied in relation to the overall SAS diagnosis of the patient. It is frequently discussed that the longer duration of an apnea/hypopnea event is a property that indicated the severity of the episode and that therefore it could be used as a factor for diagnosis. The distribution of the longer in time apnea/hypopnea event for each patient is illustrated in Fig. 2_supp (a) where the patients are grouped in the four categories of the SAS severity. Similarly, the mean duration of all apneas/hypopneas exhibited by each patient are used in the second histogram of the Fig. 2_supp (b).


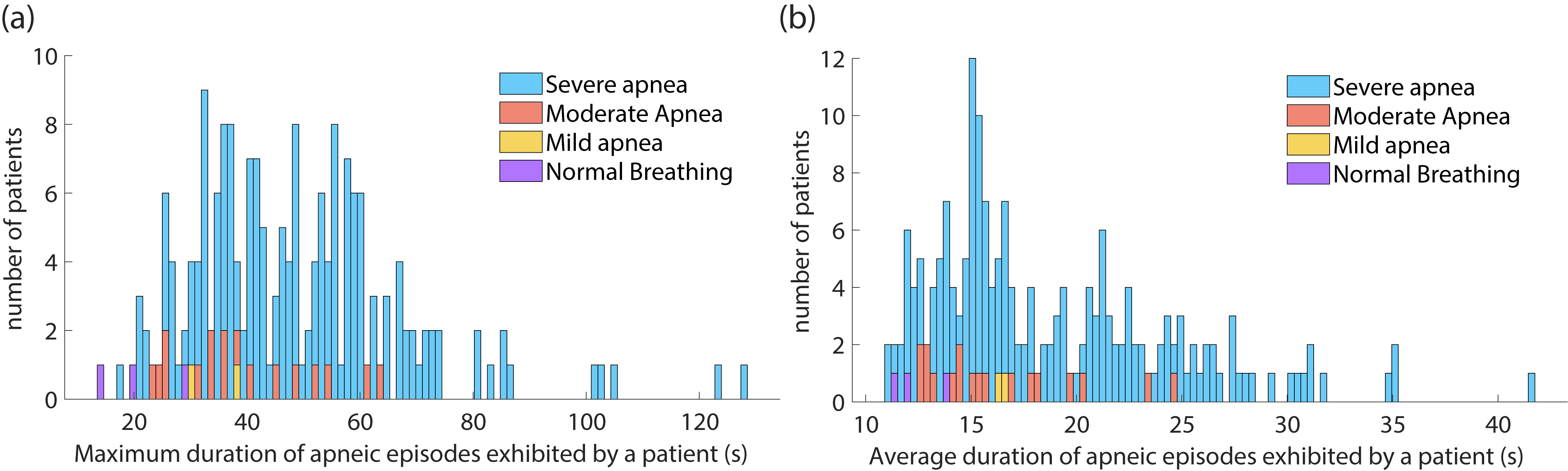


Fig. 2_supp (a) The maximum value and (b) the mean value of apneic episodes duration for each patient with regards to the overall characterization of the patient concerning the SAS severity.

# Intra-night variation of AHI for the 1^st^, 2^nd^ and 3^rd^ hour of sleep

The difference of the AHI extracted only by the first hour of sleep from the final AHI has been studied as indicative of the intra-night variability of the AHI. The same difference was extracted when the current AHI corresponds to the end of the second and the third hour of sleep. The distribution of the mean difference for all patients, illustrated in Fig. 3_supp (a-c) proves the existence of cases in which the difference remains significantly higher than the mean value, even after the 3^rd^ h of sleep.


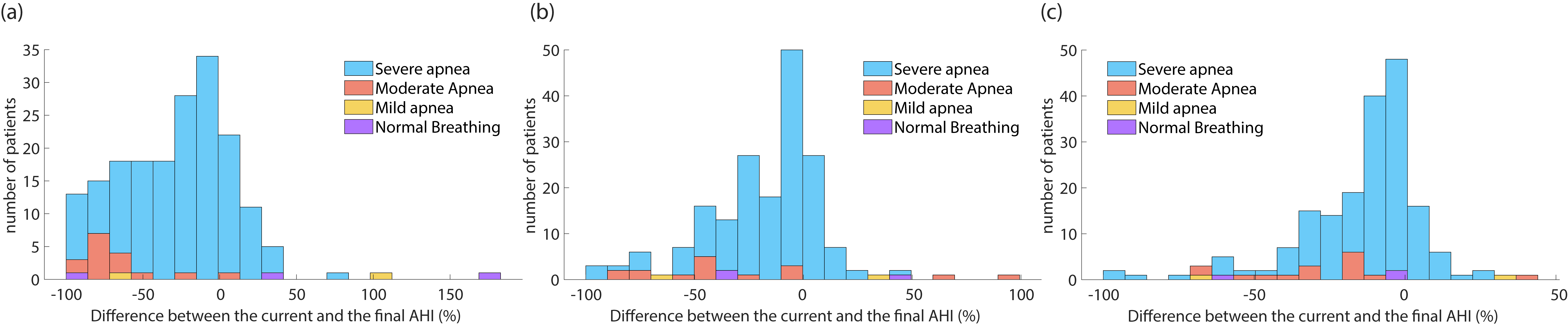


Fig. 3_supp. Distribution of patients with respect to the difference between the current and the final AHI expressed as a percentage over the final AHI, for (a) the 1^st^ hour of sleep, (b) the 2^nd^ hour of sleep and (c) the 3^rd^ hour of sleep. In these plots only the patients with at least 3 complete hours of sleep (206 out of 212) participate in the extracted statistical values.

# Apneas/hypopneas followed by oxygen desaturation event

The percentage of apneas/hypopneas followed by oxygen desaturation may be indicative of the severity of the apneic/hypopneic episode. This percentage extracted for each patient participating in the dataset is distributed over a wide range with the higher numbers representing severe patients (Fig. 4_supp (a)). However, there are patient belonging to non-apnea case (less than 5 apneas/h) whose apneic/hypopneic episodes are frequently accompanied by oxygen desaturation events (percentage > 65 %). Additionally, the time of occurrence of the relative oxygen desaturation has been studied with regards to the SAS severity of the patient (Fig. 4_supp (b)).


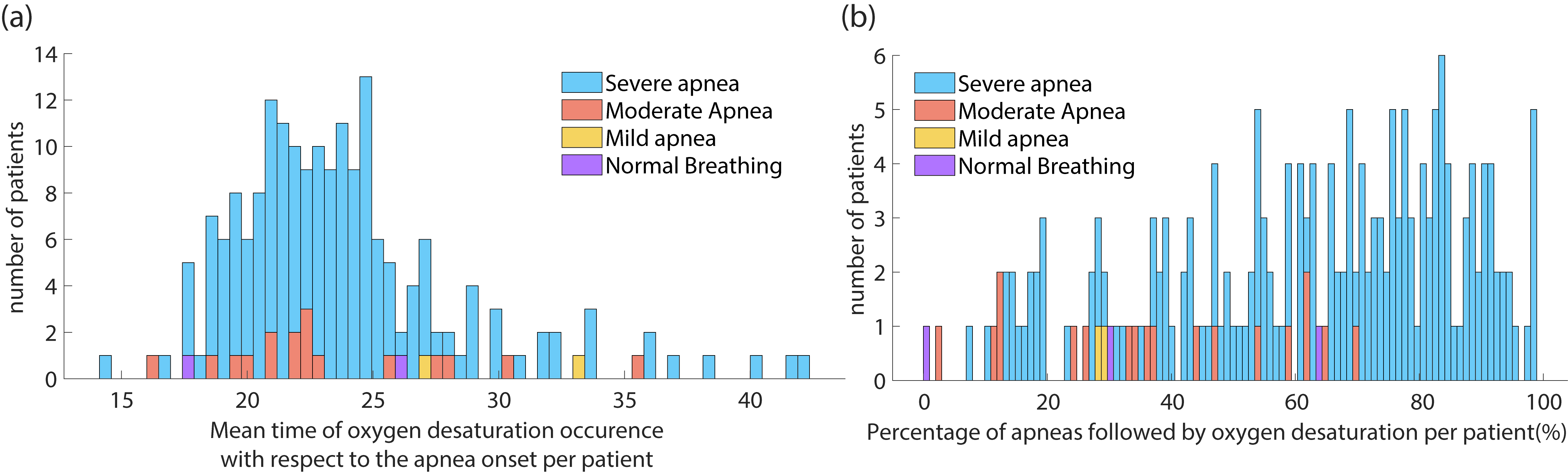


Fig. 4_supp. (a) Distribution of the percentage of apneas/hypopneas accompanied by relative oxygen desaturation per patient along with the categorization of the patients in the four SAS severity classes. (b) Distribution of the mean time of occurrence of the desaturation episodes with respect to the starting point of the associated apnea/hypopnea. The histogram presents the mean time for each patient along with the categorization of the patients in the SAS severity classes.

# Apneas/hypopneas followed by an arousal

The same process has been conducted for the arousals related to a specific apnea/hypopnea event. The results are illustrated in Fig. 5_supp (a and b).


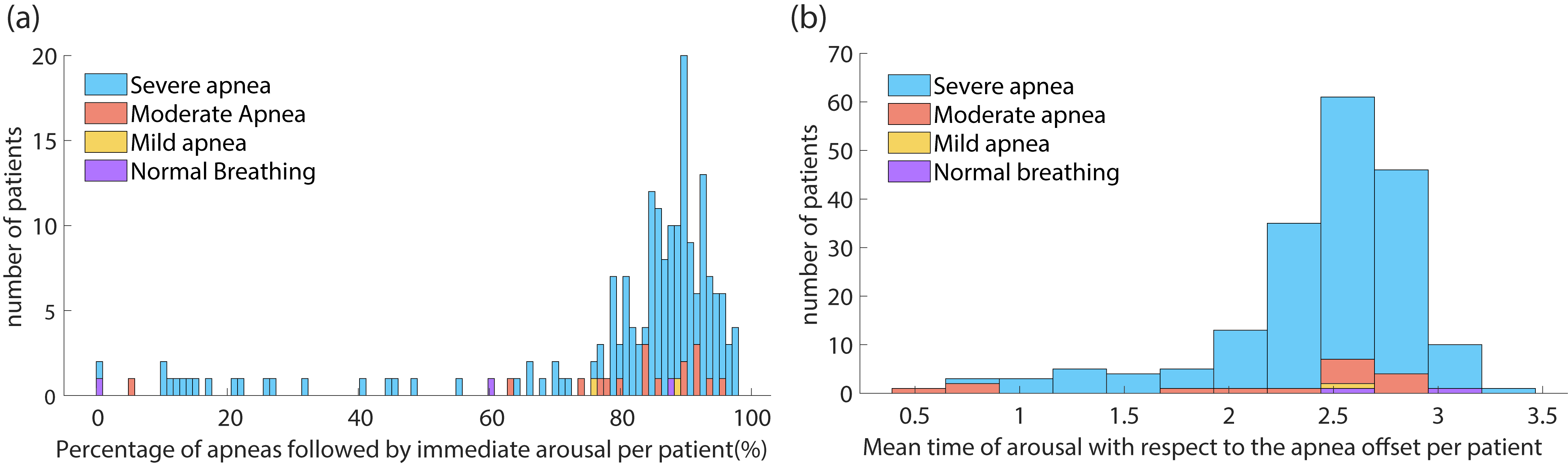


Fig. 5_supp (a) The distribution of the percentage of apneas/hypopneas that are accompanied by an immediate arousal. The distribution of the patients is based on the categorization in the four SAS severity classes. (b) The mean time of arousal with respect to the end-time of apnea/hypopnea episode per patient. The distribution of patients is accompanied by their classification in the main SAS severity categories.
